# Cyclical cholera outbreaks in Ghana: filth, not myth

**DOI:** 10.1186/s40249-018-0436-1

**Published:** 2018-06-15

**Authors:** Nana Mireku-Gyimah, Paschal Awingura Apanga, John Koku Awoonor-Williams

**Affiliations:** 1 Dansoman Polyclinic, Ghana Health Service, Greater Accra Region, Ghana; 2 Talensi District Hospital, Ghana Health Service, Tongo, Upper East Region, Ghana; 3 Policy, Planning, Monitoring and Evaluation Division, Ghana Health Service, Greater Accra Region, Ghana

**Keywords:** Cholera, Epidemic, Outbreak, Sanitation, Hygiene, Ghana

## Abstract

**Background:**

Ranked among the world’s dirtiest countries, Ghana has poor environmental sanitation and hygiene, and a lack of potable water, all of which combined have been largely blamed as the underscoring reasons for cholera outbreaks. The country has concomitantly suffered seasonal cholera outbreaks that have impacted negatively on the population’s health, as well as on the nation’s economy. To prevent cyclical cholera outbreaks in Ghana, this commentary discusses the associated problems and makes recommendations to solve them.

**Main body:**

This commentary aims to throw light on the menace of cholera in Ghana and the need to curb the recurrence of outbreaks and bouts of this epidemic. Response measures, challenges, and lessons learnt from the most recent cholera outbreak are critically assessed to determine how best this public health issue could be resolved. General and specific policy recommendations are identified in this regard.

**Conclusion:**

To resolve this problem, there is a need for an oral cholera vaccine to be introduced. There is also a need to develop strategies and interventions relating to water, sanitation, and hygiene, to be initiated by the Ministry of Health, with component activities that are culturally tailored to Ghanaian communities. Policy change towards the prevention of outbreaks in Ghana is identified as another requisite.

**Electronic supplementary material:**

The online version of this article (10.1186/s40249-018-0436-1) contains supplementary material, which is available to authorized users.

## Multilingual abstract

Please see Additional file 1 for translations of the abstract into five official working languages of the United Nations.

## Background

The dramatic consequences of cyclical cholera outbreaks in recent years in Ghana are worrying. Cholera, unlike other diarrheal diseases, is fatal even among healthy young adults, and poses a greater risk of death among immunocompromised individuals and malnourished children. The epidemics’ endemic nature in Ghana has been attributed to a lack of potable water supply, the presence of slums, unsanitary practices and poor personal hygiene, indiscriminate waste disposal, as well as street vending of contaminated water or food [1–3].

Ghana has continued to record intermittent cholera outbreaks since the 1980s, with these becoming increasingly frequent in recent years. The most recent, devastating outbreak that occurred in June 2014 spilled over to 2015. As of 4th January 2015, a total of 28 922 cases including 243 deaths were recorded, representing a case fatality rate of 0.8% [4, 5]. The outbreak affected 130 districts out of the 216 districts in all 10 regions of Ghana [5]. Until 2014, the highest number of cholera cases recorded in a single year during an outbreak was 15 032, reported in 1982 [6]. During the 2014 outbreak, the proportion of cholera cases recorded was highest among those aged 20–49 years, representing 70% of the cases.

More than 50% of the Ghanaian populace, according to a report from Global Communities [7], resides in high-density communities bordering city centers, and these communities are most prone to and hardest to control with regards to cholera outbreaks. This is because people who become unknowingly infected rapidly transmit the disease to other people who they come into contact with, making containment of the disease difficult [7]. The majority of the working force in the country falls within the age group of 20–49 years, and this reflects the economic burden that cholera imposes in Ghana [8]. It is therefore not surprising that Water and Sanitation Program [9] revealed that an estimated amount of more than 250 million US dollars (USD) is lost by the country due to premature deaths, productivity losses, and healthcare provision as a result of poor sanitation and diarrheal diseases such as cholera.

## Critique of current policy

### Response measures

Response measures during the 2014 cholera outbreak were taken at the international, national, and regional levels. The World Health Organization (WHO) provided technical support and logistics to the Ministry of Health and the Ghana Health Service towards curbing the cholera outbreak [10]. The Ghana Health Service at the national level sent cholera outbreak alerts to all regions and districts in the country. Standard operating procedures on cholera surveillance and case management were also sent to all regions. Medications and other logistics were dispatched to affected outbreak areas, and the National Cholera and Emergency Preparedness and Response Plan was updated and put into action [10].

All districts were alerted at regional levels on the importance of intensifying surveillance on diarrheal diseases. Public health messages on cholera prevention and control measures were disseminated through some media outlets in the regions [7]. Cholera cases were managed at health facilities in line with national protocols. Contact tracing was done and prophylactics were administered. District health management teams (DHMTs) in liaison with the district assemblies of Ghana provided education to districts on hygiene and sanitation.

### Challenges

While these response measures were effective to some extent in the containment of the outbreak, they were inadequate considering the large numbers of cases and vast areas that were implicated. There were inadequate hospital beds and supplies, and the number of trained volunteers was not commensurate with the extent of the outbreak. This situation largely affected the case management of individuals who were diagnosed with cholera [11].

It is instructive to note that the underlying conditions that perpetuate the recurrence of cholera outbreaks in Ghana continue to persist. Most of the people who were affected by outbreaks had poor attitudes and perception towards the disposal of refuse. Some households lacked toilet facilities, as well as proper waste disposal sites and equipment. There were also inadequate personnel responsible for waste disposal [12]. For example, Accra, the capital of Ghana, which was hardest hit during the recent outbreak, has poor sanitary conditions with deplorable drainage systems, indiscriminate disposal of waste, and large amounts of uncollected refuse in central waste bins. It was also common to see people engaging in open defecation in gutters and along beaches, or the throwing of rubbish onto the streets by pedestrians and from moving vehicles [13]. The Accra Metropolitan Assembly attributed the situation to a number of factors including poor conceptualization of sanitation, inadequate sanitary facilities, irresponsible behavior, lack of budgetary allocation towards sanitation, and the continuously increasing numbers of hawkers, indiscriminate squatters, and unauthorized and poorly located structures in the city [14].

The new government has created a Ministry of Sanitation, which may be seen as laudable. However, despite efforts made by Ghana to prevent recurrent outbreaks of cholera, concrete measures are yet to be seen. The Joint Monitoring Programme Report by the WHO and the United Nations Children’s Fund ranked Ghana as the seventh filthiest nation in the world in 2015; the country performed worse than the previous year when it was ranked tenth. It was also revealed that one in every five Ghanaians engages in open defecation due to a lack of toilet facilities, whilst only one in every eight Ghanaians regularly washes their hands [14]. Many studies have reported unhygienic practices among food handlers, including catering services, vendors, and restaurants, to be below the prescribed standards. In addition, bylaws established to scrutinize food handlers as per hygiene and sanitation standards have not been properly enforced [15–17].

## General policy recommendations

That response measures to prevent recurrence of cholera epidemics in Ghana require a multisectoral approach, which should also encompass the cooperation and participation of the public, is a known fact. The Ministry of Health in collaboration with the Ministry of Sanitation and Water Works, Ministry of Local Government and Rural Development, Ministry of Works and Housing, Ministry of Communications, Ministry of Education, municipal and district assemblies, and the Food and Drugs Authority should recognize and prioritize cholera epidemics as a crucial public health problem. This will enable them find solutions to inadequate supplies of potable water, presence of urban slums, and the enforcement of bylaws on food hygiene and environmental sanitation, as well as proper disposal of waste matter.

It is also important to intensify mass education on the prevention and control of cholera in order to improve knowledge and attitudes about the disease in communities. This should encompass the causes of the disease, the need to avoid contaminated food and water, good personal hygiene practices, and techniques for combating unsanitary practices. This public health campaign should be communicated through all available media outlets including both traditional and non-traditional channels to ensure maximum coverage.

Incentives and resources for health staff should be provided to strengthen cholera surveillance and laboratory confirmation, and case management. Budgetary allocations should also be made to ensure the availability of adequate medical supplies and logistics at health facilities for appropriate management of cholera cases. Despite the short duration of protection of the cholera vaccine, an average of three years, Ghana should also adopt the oral cholera vaccine as a preventive and control measure. This vaccine could be integrated into the country’s Expanded Program on Immunization (EPI) to ensure wide coverage, as the program operates to provide nationwide coverage of vaccine-preventable diseases. It is hoped that these recommendations will help inform policy- and decision-makers towards implementing measures that will prevent the recurrence of cholera outbreaks in Ghana.

## Specific policy recommendations

A water, hygiene, and sanitation (WASH) intervention tailored to the Ghanaian culture and initiated by the Ministry of Health can reduce the health inequities that exist in the country by improving access to toilet facilities and potable water, and by encouraging positive attitudes relating to personal and public hygiene. This initiative should help improve rural as well as peri-urban community health via the provision of adequate and potable water, proper sanitation facilities, and complementary health promotion relating to hygiene [18]. This initiative should help local Ghanaian communities by providing local agency capacity-building, community support, and physical infrastructure, as well as the promotion of behavioral change [17]. Water and sanitation facilities must be made available through this initiative to communities, including their schools and clinics. Potable water can be provided to numerous people through the construction of boreholes, piping systems for small towns, and water kiosk development where safe water can be purchased. Sanitation within communities would also be improved through household latrine construction and institutional latrine construction, as well as through the promotion of hygienic behaviors [19]_._ The component activities and expected outcomes of this WASH intervention have been selected based on the environmental and sanitation situation of the country and specifically targeted to avert cholera outbreaks in the Ghanaian setting.

The previous Ghana WASH (GWASH) program funded by the United States Agency for International Development (USAID) together with its partners was a four-year program (2009–2013) that was launched in 160 communities across five regions in the country. This program was found to be successful in improving sanitation and attitudinal change in these communities. The project was able to: leverage funds to increase access to better sanitation and water infrastructure; generate innovative methods for improving sanitation; manage and develop partnerships that foster program sustainability; and support the development of attitudes and behaviors that promote personal hygiene and enhanced sanitation [20]. The success of this program could be used to project the success of the recommended WASH program.

Priority areas for consideration of these recommendations could be informed by identification through mapping of the hardest hit communities during previous outbreaks as well as records of the most environmentally unkempt and unsanitary areas in the country. This can be achieved by use of sophisticated applications such as the geographical information system, as well as data collection and analysis from departments such as the Environmental Protection Agency, Food and Drugs Authority, Ministry of Sanitation, and district and metropolitan assemblies.

## Implementation

Implementation of the intervention would take place in community settings to ensure maximum coverage and successful implementation. In the community setting, community members, community leaders, role models, and religious leaders could be engaged and educated on how diarrheal diseases attributable to unsanitary practices and poor hygiene are impacting child morbidity and mortality among community households, and why it is imperative to prevent the disease by adopting prescribed hygienic practices, the use of safe water, and the avoidance of unsanitary behaviors. Community members could be trained to educate other community members in this regard. Durbars, storytelling sessions, religious meetings, and school curricula could incorporate education on WASH to ensure wide coverage. Initial engagement with the community will enable messages to be tailored to be culturally relevant and acceptable as per the values of the community so as to achieve successful behavioral and attitudinal change. The Mobilize, Assess, Plan, Implement, Track (MAP-IT) framework [21] would be invaluable to the implementation, monitoring, and evaluation of this public health intervention within communities. The MAP-IT framework is a systematic model that enables public health personnel to improve community health via programs tailored to targeted communities. The MAP-IT framework is appropriate for community health interventions as it involves all stakeholders and promotes community support and ownership; assesses resources available against priority needs and considers ways to use assets to meet needs; and allows for intermediary refinement through monitoring and evaluation which is incorporated from onset. This would enable the education campaign as well as the construction of water and sanitation facilities to be effective. The use of MAP-IT would also allow for quantification of the positive impact of the WASH intervention and enable the determination of aspects of the intervention that work and those that do not. In the healthcare setting, parents could be offered education through health talks, conferences, and interpersonal communication. Pamphlets and brochures could be distributed and picturesque posters could also be posted around in health facilities to educate parents on WASH. Data could also be gathered from health records and studied to determine the impact that the WASH program is having on the incidence of diarrheal diseases attributable to poor hygiene and sanitation, and to provide evidence-based advice on what strategies may be most effective to address the problem [22].

In the short term, the WASH intervention could be implemented in the most deprived communities to ensure increased numbers of access points for clean water, increased hand-washing practices among community members, and increased toilet facilities accessible to community members. The program could then be expanded in the medium and long terms to decrease the use of unclean water for daily activities, increase the awareness and knowledge of the need for environmental sanitation and its relationship with infectious disease prevention, and encourage the shunning of unsanitary practices such as open defecation. With the introduction of the oral cholera vaccine, vaccination could in the short term be carried out in response to bursts of the epidemic so as to improve the control of disease outbreaks. Vaccination could then be incorporated in the medium and long terms into the EPI to ensure mass coverage and ultimately nationwide coverage.

The budget in Table 1 provides a summary of how much money is required for the WASH program, how this money could be allocated, as well as the implementation costs involved per community. A total amount of approximately 30 000 USD per annum is projected to be required for the efficient implementation of the WASH program. This budget will enable stakeholders to have a preconceived idea of what fiscal allocations per community are necessary for the successful running of the program.

**Table 1:** Budget for the Ghana water, hygiene, and sanitation program

Budget for entire proposed project period, in USD (Direct costs only)					
Budget category totals	Initial budget period *(from Form Page 4)*	2nd additional year of support requested	3rd additional year of support requested	4th additional year of support requested	5th additional year of support requested
Personnel: *Salary and fringe benefits.*	219 000	229 000	219 000	200 000	210 000
Consultant costs	50 000	55 000	57 000	60 000	62 000
Equipment	8000	10 000	14 000	18 000	20 000
Supplies	2000	2100	2150	2200	2250
Travel	1000	1200	1300	1500	1600
Inpatient care costs	NA	NA	NA	NA	NA
Outpatient care costs	NA	NA	NA	NA	NA
Alterations and renovations	NA	NA	NA	NA	NA
Other expenses	500	700	900	1000	1200
Direct consortium/contractual costs	NA	NA	NA	NA	NA
Subtotal direct costs	280 500	298 000	294 350	282 700	297 050
F&A consortium/contractual costs	NA	NA	NA	NA	NA
Total direct costs	280 500	298 000	294 350	282 700	297 050

F&A consortium/contractual: Facilities operation and maintenance costs, depreciation, and administrative expenses are examples of costs that usually are treated as F&A costs. Budget template adopted from Laureate Education, Inc. (Executive Producer); NA: Not applicable

## Conclusions

The cyclical nature of the cholera epidemic in Ghana is a canker that requires prompt action in order to elevate the country’s public health to internationally acceptable standards. A WASH intervention uniquely formulated with component activities that will address the specific needs of the targeted communities, in a manner that will be most effective and efficient, is identified to be most likely successful. Prioritization of the recommendations outlined towards policy decisions that will address the sanitation situation in Ghana, to reduce the mortality and morbidity associated with cholera outbreaks, is imperative in this regard.

## Additional file

Additional file 1: Multilingual abstract in the five official working languages of the United Nations. (PDF 629 kb)

## Abbreviations

DHMTs: District health management teams
EPI: Expanded Program on Immunization
GWASH: Ghana water, sanitation, and hygiene
MAP-IT: Mobilize, Assess, Plan, Implement, Track
USAID: United States Agency for International Development
USD: US dollars
WASH: Water, sanitation, and hygiene
WHO: World Health Organization
