# Supplementary material for: Cyclical cholera outbreaks in Ghana: filth, not myth
Source: Infect Dis Poverty. 2018 Jun 15;7:51. doi: 10.1186/s40249-018-0436-1 (PMC6003169; doi:10.1186/s40249-018-0436-1)
Supplement: Supplementary file 1 — Multilingual abstract in the five official working languages of the United Nations. (PDF 629 kb) [file 40249_2018_436_MOESM1_ESM.pdf]

Translation of the abstract into the five official working languages of the United Nations

حالات التفشي الدوري لوباء الكوليرا في غانا: القذارة وليست الأسطورة

نانا ميركو-غيماه، باسكال أونجورا أبانغا، جون كوكو أوونوروليامز

الملخص

المعلومات الأساسية:تُصنَّف غانا من بين أقذر الدول في العالم، فهي لديها أفقر المرافق الصحية البيئية والنظافة الشخصية وتفتقر أيضًا إلى المياه الصالحة للشرب، وتُعد كل هذه الظروف مُجتمعة الأسباب الرئيسية لتفشى الكوليرا. وقد عانت البلاد في الوقت نفسه من تفشي الكوليرا الموسمية التي أثرت تأثيرًا سلبيًا على الصحة العامة للسكان بالإضافة إلى اقتصاد البلاد. وللوقاية من تفشي الكوليرا الموسمية في غانا، يتناول التعليق الوارد أدناه المشكلات المصاحبة ويقدم اقترحات لحلها.

المتن:يهدف هذا التعليق الوارد تسليط الضوء على خطورة الكوليرا في غانا والحاجة إلى منع حدوث هذا وتناوب هذا الوباء. وتساعد تدابير الاستجابة والتحديات والدروس المستفادة من تفشي الكوليرا الأخير على إجراء تقييم دقيق لتحديد أفضل طريقة لحل هذه المشكلة الصحية العامة. وفي هذا الصدد، قُدِّمت توصيات عامة وخاصة للسياسة.

النتائج: لحل هذه المشكلة، يجب تناول لقاح للكوليرا يؤخذ عن طريق الفم. وهناك أيضًا حاجة لتطوير الإستراتجيات والتدخلات المتعلقة بالمياه والمرافق الصحية والنظافة الشخصية التي يجب أن تبدأها وزارة الصحة، بالإضافة إلى أنشطة فرعية تراعي ثقافة المجتمعات الغانية. ويُعد تغير السياسة تجاه الوقاية من تفشي الوباء في غانا مطلبًا أساسيًا آخر.

Translated from English version into Arabic by Eman Shahen, proofread by Tarek Salem, through

TRANSLATORS
WITHOUT BORDERS

加纳地区周期性暴发霍乱：源于卫生条件，而非神话

Nana Mireku-Gyimah, Paschal Awingura Apanga, John Koku Awoonor-Williams

摘要

引言：加纳是全世界卫生条件最差的国家之一，较差的环境卫生和个人卫生条件以及缺乏饮用水被认为是该地区霍乱暴发的主要原因。同时加纳还需应对季节性霍乱疫情，霍乱疫情严重影响了人民身体健康和国家经济。为了防止周期性霍乱疫情的发生，本文将讨论相关问题，并提出解决问题的建议。

主要内容：本文旨在揭示加纳地区遭受的霍乱威胁，以及遏制该病流行和再次暴发的必要性。对最近爆发的霍乱疫情所采取的应对措施，面临的挑战及经验教训进行严格评估，以确定应该如何最大限度地解决公共卫生问题，在此基础上，确定全面和具体的政策建议。

结论：为解决该地区周期性霍乱疫情，需要引进口服霍乱疫苗，同时需要由卫生部启动与 WASH 相关的战略和干预措施，其中包括针加纳社区的文化活动。将政策转向如何预防霍乱暴发是另一个重要措施。

Translated from English version into Chinese by Xue-Jiao Teng, edited by Pin Yang

CDC CHINESE CENTER FOR DISEASE CONTROL AND PREVENTION
NATIONAL INSTITUTE OF PARASITIC DISEASES

Épidémies cycliques de choléra au Ghana: saleté et vérité

Nana Mireku-Gyimah, Paschal Awingura Apanga, John Koku Awoonor-Williams

Résumé

Contexte: Classé parmi les pays les plus sales du monde, le Ghana souffre d'un mauvais assainissement de l'environnement, d'une mauvaise hygiène et d'un manque d'eau potable, trois facteurs combinés auxquels les flambées de choléra sont largement imputables. Le pays a subi plusieurs flambées saisonnières de choléra simultanées, qui ont a eu des effets négatifs sur la santé de la population ainsi que sur l'économie du pays. Afin de prévenir les épidémies cycliques de choléra au Ghana, cet article discute des problèmes associés et formule des recommandations visant à les résoudre.

Discussion: Cet article vise à faire la lumière sur la menace du choléra au Ghana et sur la nécessité de freiner la répétition des flambées et des épisodes épidémiques. Les moyens de riposte, les difficultés et les enseignements tirés de la flambée de choléra la plus récente sont analysés de façon critique afin de déterminer quelle serait la meilleure manière de régler ce problème de santé publique. Des recommandations de politiques générales et spécifiques sont identifiées dans ce but.

Conclusion: Afin de résoudre ce problème, il est nécessaire d'introduire un nouveau vaccin anticholérique oral. Il y a également besoin d'élaborer des stratégies et des interventions par rapport à l'eau, à l'assainissement et à l'hygiène, qui doivent être engagées par le ministère de la Santé en les adaptant du point de vue culturel aux populations ghanéennes. La réorientation de la politique vers la prévention des flambées au Ghana est considérée comme un autre prérequis.

Translated from English version into French by Suzanne Assenat, proofread by Gladis Audi, through

TRANSLATORS WITHOUT BORDERS

Периодические вспышки холеры в Гане: грязь без прикрас

Нана Миреку-Джима, Паскаль Авингура Апанга, Джон Коку Авунор-Уильямс

Аннотация

Контекст исследования: Плохие санитарно-гигиенические условия и нехватка питьевой воды делают Гану одной из самых грязных стран мира, а в совокупности эти факторы считаются основными причинами вспышек холеры в стране. Наряду с этим в Гане наблюдаются сезонные вспышки холеры, которые наносят вред как здоровью населения, так и экономике страны. В данном комментарии в рамках предотвращения периодических вспышек холеры в Гане обсуждаются смежные проблемы и даются рекомендации по их устранению.

Основная часть: Цель данного комментария — пролить свет на угрозу холеры в Гане и показать необходимость пресечения повторных вспышек и эпидемий этой инфекции. Чтобы определить наиболее эффективное решение данной проблемы здравоохранения, изучается самая

недавняя вспышка холеры и проводится критическая оценка мер реагирования, основных проблем и извлеченных уроков. В связи с этим определяются общие и конкретные рекомендации по вопросам политики.
Заключение: Для решения этой проблемы необходимо начать пероральную вакцинацию против холеры. Необходимо также разработать культурно адаптированные к сообществам Ганы стратегии и мероприятия по улучшению водоснабжения, санитарии и гигиены, которые будет проводить Министерство здравоохранения. Еще одним условием для предотвращения вспышек в Гане является изменение политики.

Translated from English version into Russian by Polina Nikitina, proofread by Ann Nosova, through

TRANSLATORS WITHOUT BORDERS

Brotes de cólera cíclicos en Ghana: inmundicia, no un mito

Nana Mireku-Gyimah, Paschal Awingura Apanga, John Koku Awoonor-Williams

Resumen

Antecedentes: Clasificado como uno de los países más sucios del mundo, Ghana tiene un saneamiento y una higiene ambiental deficientes, así como también falta de agua potable. Todo esto combinado ha sido considerado, durante mucho tiempo, el motivo detrás de los brotes de cólera. El país ha sufrido simultáneamente brotes de cólera estacional que han tenido un impacto negativo en la salud de la población, así como en la economía de la nación. Para evitar brotes de cólera cíclicos en Ghana, esta observación analiza los problemas asociados y hace recomendaciones para resolverlos.
Cuerpo principal: Esta observación pretende echar luz sobre la amenaza del cólera en Ghana y la necesidad de frenar la repetición recurrente de brotes y episodios de esta epidemia. Las medidas de respuesta, los desafíos y las lecciones aprendidas del brote de cólera más reciente se evalúan críticamente para determinar la mejor forma de resolver este problema de salud pública. A este respecto, se identifican recomendaciones sobre políticas generales y específicas.
Conclusión: Para resolver este problema, es necesario introducir una vacuna oral contra el cólera. También es necesario desarrollar estrategias e intervenciones relacionadas con el agua, el saneamiento y la higiene. Estas estrategias serán iniciadas por el Ministerio de Salud, con actividades que se adapten culturalmente a las comunidades ghanesas. Otro requisito que se identifica es la necesidad de un cambio de políticas hacia la prevención de brotes en Ghana.

Translated from English version into Spanish by Ximeniuts, proofread by Alexander9696, through

TRANSLATORS WITHOUT BORDERS
